# Single crystal hybrid perovskite field-effect transistors

**DOI:** 10.1038/s41467-018-07706-9

**Published:** 2018-12-17

**Authors:** Weili Yu, Feng Li, Liyang Yu, Muhammad R. Niazi, Yuting Zou, Daniel Corzo, Aniruddha Basu, Chun Ma, Sukumar Dey, Max L. Tietze, Ulrich Buttner, Xianbin Wang, Zhihong Wang, Mohamed N. Hedhili, Chunlei Guo, Tom Wu, Aram Amassian

**Affiliations:** 1 KAUST Solar Center (KSC) and Division of Physical Sciences and Engineering (PSE), King Abdullah University of Science and Technology (KAUST), Thuwal, 23955-6900 Saudi Arabia; 2 The Guo China-US Photonics Laboratory (GPL), State Key Laboratory of Applied Optics (SKLAO), Changchun Institute of Optics, Fine Mechanics and Physics (CIOMP), Chinese Academy of Sciences (CAS), Changchun, 130033 People’s Republic of China; 3 Division of Physical Sciences and Engineering (PSE), King Abdullah University of Science and Technology (KAUST), Thuwal, 23955-6900 Saudi Arabia; 4 Nanofabrication Core Lab, King Abdullah University of Science and Technology (KAUST), Thuwal, 23955-6900 Saudi Arabia; 5 Imaging and Characterization Core lab, King Abdullah University of Science and Technology (KAUST), Thuwal, 23955-6900 Saudi Arabia; 6 The Institute of Optics, University of Rochester, Rochester, NY 14627 USA; 7 School of Materials Science and Engineering, University of New South Wales (UNSW), Sydney, NSW 2052 Australia; 8 Department of Materials Science and Engineering, North Carolina State University, Raleigh, 10, NC 27695 USA; 9 Present Address: Department of Microbial and Molecular Systems, Centre for Surface Chemistry and Catalysis, KU Leuven — University of Leuven, Celestijnenlaan, 200F, Leuven B-3001 Belgium

## Abstract

The fields of photovoltaics, photodetection and light emission have seen tremendous activity in recent years with the advent of hybrid organic-inorganic perovskites. Yet, there have been far fewer reports of perovskite-based field-effect transistors. The lateral and interfacial transport requirements of transistors make them particularly vulnerable to surface contamination and defects rife in polycrystalline films and bulk single crystals. Here, we demonstrate a spatially-confined inverse temperature crystallization strategy which synthesizes micrometre-thin single crystals of methylammonium lead halide perovskites MAPbX_3_ (X = Cl, Br, I) with sub-nanometer surface roughness and very low surface contamination. These benefit the integration of MAPbX_3_ crystals into ambipolar transistors and yield record, room-temperature field-effect mobility up to 4.7 and 1.5 cm^2^ V^−1^ s^−1^ in *p* and *n* channel devices respectively, with 10^4^ to 10^5^ on-off ratio and low turn-on voltages. This work paves the way for integrating hybrid perovskite crystals into printed, flexible and transparent electronics.

## Abstract (web summary)

The methylammonium lead halide perovskites have shown excellent optoelectronic properties but the field-effect transistors are much less studied. Here Yu et al. synthesize micrometer-thin crystals of perovskites with low surface contamination and make ambipolar transistor devices with high mobilities.

## Introduction

Hybrid organic–inorganic lead halide perovskites (CH_3_NH_3_PbX_3_, X = Cl, Br, and I) have emerged as a remarkable solution-processable semiconductor over the past few years, fast becoming a serious contender for efficient thin film photovoltaics (PVs)^1–3^. Good carrier transport properties along the vertical direction have made even polycrystalline films with small grain size sufficiently good to achieve power conversion efficiency of more than 20%. The situation is markedly different in electronic devices, such as field-effect transistors (FETs), where fast and unimpeded lateral charge transport should occur through the confines of the semiconductor–dielectric interface over much longer distances^4,5^. Consequently, the presence of grain boundaries, interfacial contamination, and other defects can severely hinder field-effect carrier mobility and FET device operation^6,7^. Grain boundaries in polycrystalline hybrid perovskites have been linked to significant lateral tunnel junctions, a problem further exacerbated by the tendency of lead halide and perovskite hydrates to form at surfaces, interfaces, and even in grain boundaries^8–10^. It is perhaps for these reasons that very few reports have emerged on working perovskite FETs, particularly at room temperature^7,11–19^. Duan and co-workers^11–13^ made CH_3_NH_3_PbI_3_ FETs with different size microplates and demonstrated field-effect mobility at low temperatures. Sirringhaus and co-workers^14^ optimized thin film processing and achieved room temperature electron field-effect mobility of 0.5 cm^2^ V^−1^ s^−1^. More recently, Nazeeruddin and co-workers^15^ demonstrated high quality mixed cation perovskite films yielding balanced ambipolar FETs with carrier mobility ca. 2 cm^2^ V^−1^ s^−1^ at room temperature.

Single crystals (SCs) are normally far less defective than polycrystalline films in most materials and may be best suited to overcome these challenges, as they eliminate grain boundary defects altogether. Bulk SCs (BSCs) of hybrid perovskites have already been reported to exhibit higher carrier mobility, longer carrier diffusion length, and lower trap density than polycrystalline films^20,21^. To date, however, bulk perovskite SCs continue to underperform in optoelectronic devices, such as solar cells^21–23^ and photodetectors^22,24^, as compared to their polycrystalline thin film (PTF) counterparts^25–28^, and there are no reports to date of SC perovskite use in FETs. This can be attributed to extensive surface contamination due to incomplete precursor conversion and hydration of the perovskite crystal faces, which change the near-surface composition, structure, morphology, and electronic properties^9,22,25,29^. Such imperfections are expected to severely impact electrical contacts, interfacial charge transport in the channel and to hurt FET device operation^11,28^. Cleaning and peeling procedures have been attempted and are commonly used prior to investigation of optoelectronic properties of BSCs, but these are not practical and have not been shown to work for FET devices^21,22^. Integration of crystals into planar device architectures is also challenging unless crystallization methods, such as anti-solvent vapor-assisted crystallization^20^, low temperature solution^21,22^, or inverse temperature crystallization approaches^30,31^ are adapted to the device integration needs as well.

Here, we show a spatially confined inverse temperature crystallization method that successfully synthesizes controlled micrometer-thin single crystals (TSCs) of different hybrid perovskites (MAPbX_3_, X = Cl, Br, and I) with tunable lateral size ranging from micrometers to millimeters, as needed. As confinement templates the crystal’s top and bottom facets, the SC achieves conformal growth on flat and pre-patterned surfaces with excellent adhesion. TSCs with sub-nanometer surface roughness and remarkably low surface contamination are demonstrated despite fabrication in ambient air. Ambipolar MAPbX_3_-based TSC-FETs with bottom-gate bottom-contact and bottom-gate top-contact architectures are demonstrated and achieve record field-effect hole (electron) mobilities as high as 3.8 (0.32), 3.6 (0.26), and 4.7 (1.51)cm^2^ V^−1^s^−1^ for X = Cl, Br, and I, respectively. The devices also exhibited on/off ratio up to 10^5^ and low turn-on and threshold voltages. Substrates pre-patterned with Au contacts were also shown to yield working FETs, demonstrating conformal crystal growth on patterned surfaces. The successful demonstration of high-performance TSC-FETs is attributed to the superior semiconductor–dielectric interface in the channel achieved owing to the significantly reduced roughness, defects, and contamination of the confined crystal facet as compared to polycrystalline films and free-grown BSCs. This approach paves the way for electronic devices and circuits based on hybrid perovskite semiconductors and also addresses key integration issues for perovskite single crystals into devices requiring lateral charge transport.

## Results

### Fabrication of hybrid perovskite TSCs

**Fig. 1:**
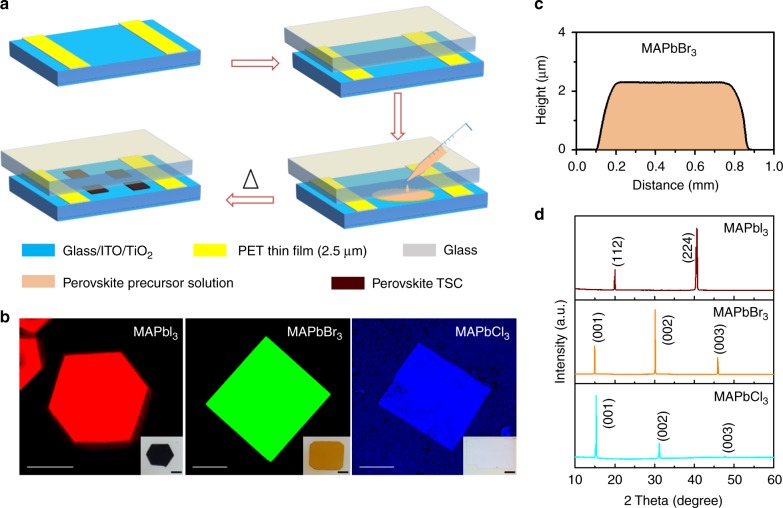
Hybrid perovskite TSC fabrication. **a** Schematic representation of spatially confined inverse temperature crystallization method for producing thin single crystals (TSCs). **b** Fluorescence microscopy images of MAPbI_3_, MAPbBr_3_, and MAPbCl_3_ TSCs (which are excited with a pulsed 450, 473, and 405 nm laser, respectively). Scale bar: 100 μm. Inset: optical images of MAPbI_3_, MAPbBr_3_, and MAPbCl_3_ TSCs. Scale bar: 200 μm. **c** Height profile of MAPbBr_3_ TSC indicating its thickness is about 2.45 µm. **d** XRD spectra of synthesized MAPbX_3_ TSCs, where X = I, Br, and Cl, respectively

Saidaminov et al.^30^ first reported that MAPbX_3_ (X = Cl, Br, and I) perovskites exhibit an inverse temperature solubility behavior in certain solvents, which has been utilized to grow macroscopic perovskite single crystals^32,33^. Liu et al.^31^ developed the technique and fabricated 150-µm-thick single-crystalline perovskite wafers. Chen and co-workers.^23^ demonstrated that thickness can be tuned by changing the distance between two substrates. Our method takes a further step in the direction of TSC fabrication by confining the starting solution between two substrates—one or both of which can be pre-patterned with electrodes—separated by a fixed spacer intended to control the thickness, contact quality, and promote lateral growth. In Fig. 1a, we show a schematic representation of the thickness-confined crystallization strategy. In this example, a 2.5-µm-thick polyethylene terephthalate (PET) film was used as spacer between two glass slides and molten at 270 °C to achieve good adhesion. The perovskite ink was injected between the two slides and was drawn through capillary forces. Crystallization occurred under heating (see Methods for details). A key difference between this approach and the inverse temperature crystallization in a beaker, vial, or even a substrate^30^ is that our method provides additional control knobs of importance to device integration, in terms of the confinement spacing, geometry, choice of confining materials—including surface engineered or pre-patterned surfaces, and the starting volume of ink, which can be replenished as needed. The confinement spacer and the volume of ink determine the thickness and lateral size of the TSCs, respectively, while the confining material also dictates the roughness of the crystal’s top and bottom surfaces. Fluorescence microscopy images and related optical micrographs of typical MAPbX_3_ (X = Cl, Br, and I) crystals grown using this method (Fig. 1b) show lateral dimensions reaching the millimeter scale in a single run, while the thickness (approximately 2.45 μm) is fixed by the spacer (Fig. 1c, Supplementary Figure 1a–c).

### Single crystal microstructure and surface properties

**Fig. 2:**
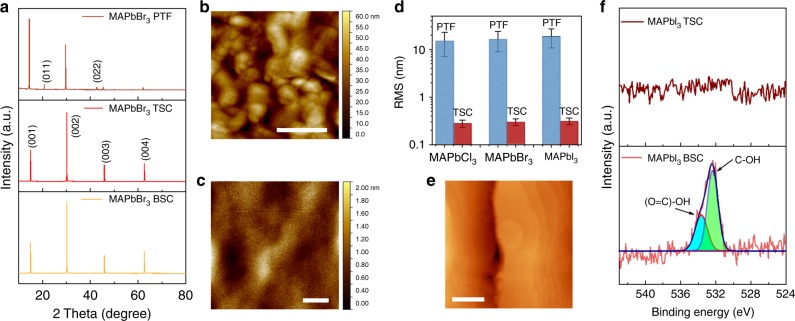
Microstructure and surface properties of TSCs. **a** XRD data comparing the polycrystalline thin film (PTF) to the TSC and bulk single crystal (BSC) forms of MAPbBr_3_. Surface topography of a MAPbBr_3_ PTF (**b**) and that of a TSC (**c**) as measured by AFM, of which the root mean squared (RMS) roughness for PTF and TSC are 16.5 and 0.21 nm, respectively. Scale bar: 400 nm. **d** RMS surface roughness of MAPbX_3_ (X = Cl, Br, and I) polycrystalline film and TSCs. The error bar reflects the statistical variation in roughness in different parts of the sample. **e** Scanning tunneling microscopy (STM) image of the surface region near the edge of a MAPbBr_3_ TSC. Scale bar: 50 nm. **f** XPS spectra of the surface of MAPbI_3_ TSC and BSC

Notable advantages of the confined crystallization method are the superior microstructure and morphology in comparison with polycrystalline films. These advantages include formation of a single domain macroscopic crystal that is free of domain and grain boundaries and which can be defined as a single crystal and behave as such. We confirm this by inspecting the crystalline structure, optical properties, and surface morphology of the TSCs. X-ray diffraction (XRD) measurements on MAPbX_3_ TSCs (Fig. 1d) reveal that the MAPbCl_3_ and MAPbBr_3_ TSCs have a cubic lattice, while the MAPbI_3_ TSC has a tetragonal lattice. XRD data comparing PTFs, TSCs, and BSCs of MAPbBr_3_ (Fig. 2a) clearly show that the structure of the TSC is identical to BSC samples, as expected. We further analyzed the crystallinity of the MAPbBr_3_ TSC using XRD rocking curve (XRC) analysis of the (001) diffraction (Supplementary Figure 2). The full-width at half-maximum was found to be 0.063°. The narrow width of the (001) peak is quantitatively comparable to the other reported values for MAPbBr_3_ SCs and validates the high-quality single-crystalline nature of our materials^31,34,35^. Ultraviolet–Visible (UV–Vis) absorbance and photoluminescence spectra of the TSCs are also very similar to those of BSCs (Supplementary Figure 1d, 1e).

Importantly, the surface of the TSCs look featureless in the confined regions under scanning electron microscopy (SEM, Supplementary Figure 3) and atomic force microscopy (AFM, Fig. 2b, c). By contrast, PTFs exhibit a large number of crystals, domains, and pinholes, while the surface of the BSC shows evidence of significant corrugation and roughness left over from the crystallization process. The surface of the TSC inspected after removal of the confining superstrate is extremely smooth, as well as free of any apparent grain and domain boundaries, pointing to their single-crystalline nature. AFM images comparing MAPbBr_3_ thin film (Fig. 2b) and TSC (Fig. 2c) surfaces reveal a starkly different root mean-squared (RMS) surface roughness of 16.5 nm vs. 0.21 nm, respectively. In fact, we found the RMS roughness of all three halide TSCs to be one to two orders of magnitude lower than polycrystalline films and bulk crystals (Fig. 2d)^21,29,30,36–39^. This is largely owing to the confined growth method which forces the crystal to undergo conformal growth against the very smooth confining substrate and superstrate.

Further examination of the surface of TSCs revealed the appearance of atomically flat terraces near the unconfined edges of the crystals, as seen by scanning tunneling microscopy (STM) measurements performed in ultra-high vacuum (UHV conditions). STM micrographs obtained in UHV (Fig. 2e) reveal atomically flat terraces with an apparent step height consistent with perovskite lattice planes (crystal lattices of 5.68 and 5.92 Å for cubic MAPbCl_3_ and MAPbBr_3_, respectively). The appearance of terracing with monolayer steps on one or more of the facets of a macroscopic crystal is usually ascribed to its homoepitaxial growth behavior, either via layer-by-layer or step flow processes. This offers further evidence in favor of the single-crystalline nature of TSCs. We did not observe this layered structure on the edges of MAPbI_3_ TSCs, which may be due to the tetragonal crystal structure of MAPbI_3_ TSCs^26^, whereas the other crystals are cubic.

X-ray photoelectron spectroscopy (XPS) measurements performed on MAPbI_3_ TSCs and BSCs show clear peaks at 533.7 and 532.4 eV in the latter case, which are assigned to the –COOH^40^ and –COH groups^41^, respectively (Fig. 2f). These peaks are absent from the surface of TSCs, which is remarkable for these materials considering that the confined samples were grown in ambient air. Separation from the superstrate was performed inside a nitrogen glove box prior to the XPS measurement and transfer to UHV by using a vacuum suitcase to avoid additional contamination by exposure of the free surface to air. The XPS analysis indicates that the buried interfaces of TSCs grown via confinement are mostly free of the surface chemical contaminations commonly seen on free-grown crystals and polycrystalline films.

The formation of high-quality, planar, smooth, grain boundary-free, and contamination-free crystal facets is particularly useful in the context of device integration. Many device architectures require at least one high-quality facet to make contacts, while others may require two parallel facets. Bottom-gate bottom-contact (BGBC) and top-gate top-contact FETs require one high-quality facet for both channel and contacts, whereas bottom-gate top-contact (BGTC) and top-gate bottom-contact FETs use opposing facets for channel and contacts. Below, we will demonstrate that the confinement method can yield working BGBC and BGTC FET devices requiring one or two facets of the TSCs, respectively.

### Single crystal FETs

**Fig. 3:**
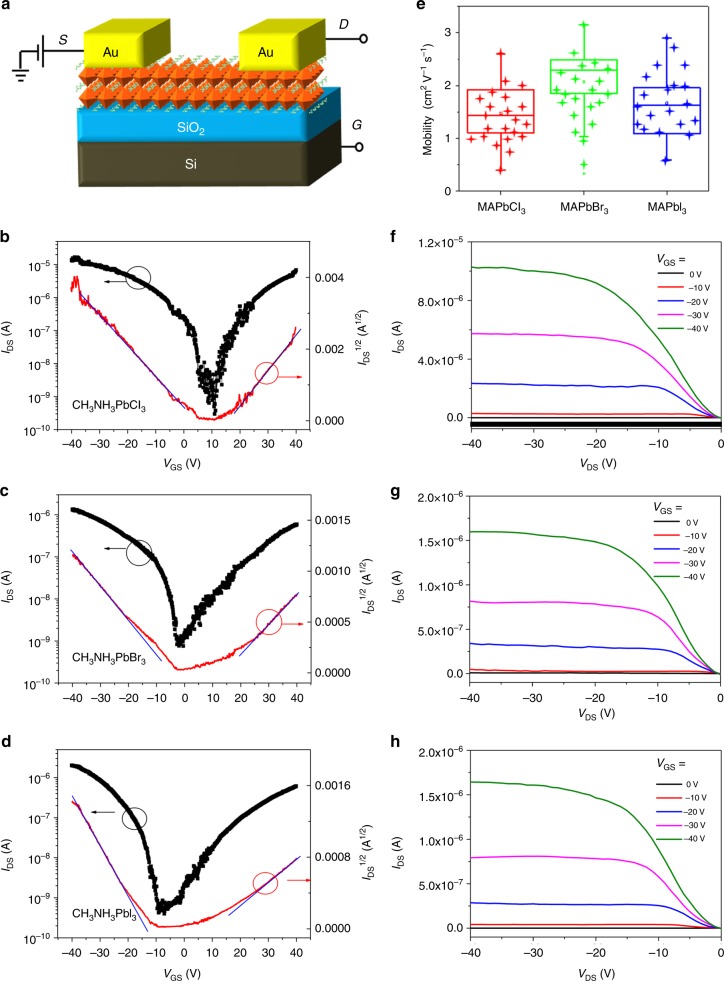
Characteristics of top-contact TSC-FETs. **a** Schematic of the bottom-gate, top-contact (BGTC) device with a TSC hybrid perovskite as semiconductor layer. Transfer characteristics *(I*_DS_ vs. *V*_GS_ (black solid square) and *I*_DS_^1/2^ vs. *V*_GS_ (red solid line)), as well as the fit lines (blue solid line) for **b** MAPbCl_3_, **c** MAPbBr_3_, and **d** MAPbI_3_ TSC-FETs, *V*_GS_ ranging from −40 to 40 V. *V*_DS_ = −30 V. **e** Saturation mobility statistics for 60 devices. The box plot graphically depicts the statistical population of numerical data, including the maximum, the minimum, as well as the average (median) mobilities. Channel length: 50 μm. Output characteristics of TSC-FETs based on **f** MAPbCl_3_, **g** MAPbBr_3_, and **h** MAPbI_3_

We have fabricated BGTC FETs with channel length (*L*) ranging from 10 to 150 µm (schematic shown in Fig. 3a). The channel width (*W*) depended upon the lateral size of the TSCs and was measured individually for each TSC that was successfully coated with a pair of Au electrodes (see Supplementary Figure 4). A highly *n*-doped Si wafer with a 240 nm SiO_2_ layer (capacitance *C*_i_ = 15nFcm^−2^) was employed as the substrate, gate electrode, and gate dielectric. We found Au (work function around 4.7 eV)^42^ to be a suitable contact for both hole and electron injection as the valance band maximum/conduction band minimum of MAPbX_3_ (X = I, Br, and Cl) are 3.8/5.3 eV for I, 3.38/5.68 eV for Br, and 2.94/5.82 eV for Cl^43^. Additional BGTC devices were also fabricated with lower work function contacts, including Ag and Al. However, the devices either did not operate or were damaged shortly after initiating measurement. We suspect chemical damage to the contacts which showed evidence of discoloration shortly after fabrication. Figure 3b–d show the ambipolar transfer characteristics of the BGTC devices based on MAPbCl_3_, MAPbBr_3_, and MAPbI_3_ TSCs measured at room temperature at a drain voltage (*V*_DS_) of −30 V for gate voltage (*V*_GS_) sweep from −40 V to 40 V, along with *I*_DS_^1/2^ vs. *V*_GS_ plots. The plots appear linear over a wide range of *V*_GS_ for both *p*- and *n*-type transports, which demonstrates ambipolar behavior and high-quality devices. It is worth mentioning that the transport property is sensitive to device fabrication conditions, such as the temperature, electrode materials, doping state, and dielectric interface^7,13,16^. In this case, the use of Au electrodes, along with room temperature measurement of device characteristics, may explain the ambipolar behavior consistent with previous reports^13,16^. The field-effect mobility (*μ*) can be extracted in the saturation regime using the saturated *I*_DS_ vs. *V*_GS_ relationship: $I_{{\mathrm{DS}}} = \frac{W}{{2L}}C_{\mathrm{i}}\mu \left( {V_{{\mathrm{GS}}} - V_{{\mathrm{TH}}}} \right)^2$. Accordingly, the maximum saturation hole (electron) mobilities for the devices based on MAPbCl_3_, MAPbBr_3_, and MAPbI_3_ TSCs are found to be 2.6 (2.2), 3.1 (1.8), and 2.9 (1.1)cm^2^V^−1^s^−1^, respectively (see Table 1 and Supplementary Table 1). Supplementary Figure 5 shows the saturation field-effect mobility vs. gate voltage for the devices based on MAPbCl_3_, MAPbBr_3_, and MAPbI_3_ TSCs employing the top-contact configuration. The saturation mobility values stay flat after the tune-on gate voltage and agree with the values reported in Table 1 and Supplementary Table 1. The statistical distribution of hole mobility for 60 devices is shown in Fig. 3e, with the average hole (electron) mobility of 1.8 (1.3), 1.9 (1.1), and 1.5 (0.7)cm^2^V^−1^s^−1^, respectively, for MAPbCl_3_, MAPbBr_3_, and MAPbI_3_ TSC devices. The mobility was found to increase as the channel increased from 10 to 150μm (Supplementary Figure 6a), which may be attributed to reduced relative influence of contact resistance as the channel resistance increases (Supplementary Figure 6b–d), while field-dependent transport does not appear to be dominant. The current on/off ratios are found to be on the order of 2.4 × 10^4^, 4.8 × 10^3^, and 6.7 × 10^3^, while the threshold voltages (*V*_TH_) are found to be 4.4, −4.9, and −13.2 V, for X = Cl, Br, and I, respectively. The positive shift of *V*_TH_ may be attributed to unintentional oxygen-induced hole doping from the bottom dielectric surface^6^, which was UV-O_3_ treated to increase hydroxyl groups and enhance the adhesion between TSCs and the SiO_2_/Si substrate^11^.

**Table 1:** The figures of merit of BGTC TSC-FET devices based on MAPbX_3_ (X = Cl, Br, and I)

TSCs	*W*/*L* (µm µm^−1^)	Highest *µ*_h_ (cm^2^ V^−1^ s^−1^)	Average *µ*_h_ (cm^2^ V^−1^ s^−1^)	On/off ratio	*V*_TH_ (V)	*N*_it_ (eV^−1^ cm^−2^)	*S* (V dec^−1^)
MAPbCl_3_	265/50	2.6	1.8	2.4 × 10^4^	4.4	5.3 × 10^12^	4.3
MAPbBr_3_	240/50	3.1	1.9	4.8 × 10^3^	−4.9	4.6 × 10^12^	4.4
MAPbI_3_	185/50	2.9	1.5	6.7 × 10^3^	−13.2	3.7 × 10^12^	3.8

Statistics were based on 60 devices

The output characteristics (*I*_DS_ vs. *V*_DS_) obtained for the devices operating in the hole-enhancement mode are displayed in Fig. 3f–h. At low *V*_GS_, the device exhibits ambipolar transport with diode-like current–voltage (*I–V*) characteristics; however, at high *V*_GS_, unipolar transport was observed with standard linear-to-saturation *I*–*V* transistor characteristics. The performance of FETs is normally affected by the contact resistance between the source/drain electrodes and the active layer^44^, and the total device resistance *R*_total_ multiplied by the channel width *W* is calculated to be 80, 150, and 195kΩcm for MAPbCl_3_, MAPbBr_3_, and MAPbI_3_, respectively, (Supplementary Figure 6b–d) highlighting generally good contacts. Supplementary Figure 7 summarizes the results of the transconductance (d*I*_DS_/d*V*_GS_)^45,46^ for our perovskite SC transistors. The best-performing *p*-type device based on MAPbCl_3_ SCs possesses a peak transconductance of 0.2 mS (at *V*_GS_ = −35 V). We believe that the high carrier mobility of perovskite SCs is the key reason behind the high currents and transconductances observed. The contact resistance and transconductance provide fine perspectives to analyze the device, which are valuable for investigating the carrier injection at the interface between the perovskite and electrodes and for designing better device with large amplification^45,46^.

**Fig. 4:**
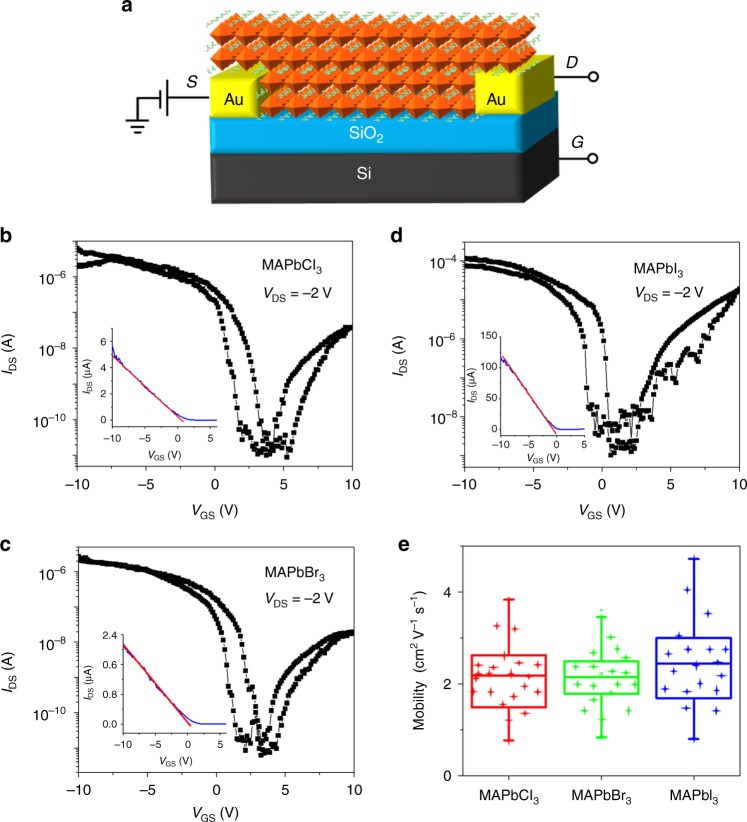
Characteristics of bottom-contact TSC-FETs. **a** Schematic representation of bottom-gate, bottom-contact (BGBC) TSC-FET device. Representative transfer characteristics of TSC-FETs of **b** MAPbCl_3_, **c** MAPbBr_3_, and **d** MAPbI_3_ using forward/reverse gate voltage sweeps from −10 to 10 V at a rate of 0.05 V s^−1^, with *V*_DS_ = −2 V. Insets of **b**–**d** show the linear regime of the respective devices. **e** Field-effect hole mobility distribution for 20 devices fabricated and tested for each halide perovskite

It is important to note that BGTC devices were successful despite charges having to travel through a micrometer-thick semiconductor layer to reach the channel, much more than is typical for thin film transistors^5,44^. To exclude the effect of crystal thickness, we have fabricated BGBC devices by growing SCs directly onto the substrate pre-patterned with Au electrodes (Fig. 4a). One key benefit of this approach is the excellent adhesion of the SCs to the pre-patterned electrodes, as indicated by delamination of the metal contacts from the substrate when attempting to peel off the crystals (Supplementary Figure 8). The bottom surface morphologies of perovskite TSCs were imaged by AFM as shown in Supplementary Figure 9, which reveals a small RMS roughness (0.70, 1.23, and 1.02 nm for MAPbCl_3_, MAPbBr_3_, and MAPbI_3_, respectively) indicative of a smooth interface for the buried interface as well. However, we also noted instances where Au contacts seemed discolored and other instances where Au contacts delaminated, suggesting undesirable chemical interactions with the perovskite ink or etching of the Cr adhesion layer^47^. Perhaps as a result of this, BGBC devices tended to fail when the applied gate voltage was >± 10 V, thus limiting the field-effect mobility analysis window to the linear regime. Not surprisingly, BGBC devices fabricated with substrates pre-patterned with Ag and Al electrodes failed to operate, as the contacts did not survive the harsh environment and conditions of crystal growth. In some cases, Au electrodes were also seen to delaminate and float to the surface of the solution after just a few hours of inverse temperature crystallization. We ascribe this to etching of the Cr adhesion layer used prior to Au deposition.

For MAPbI_3_, generally smaller crystals were typically obtained in the presence of pre-patterned Au electrodes (see Supplementary Figure 10), suggesting that the presence of metal influences the crystallization process. The transfer curves of BGBC devices based on MAPbX_3_ with X = Cl, Br, and I (Fig. 4b–d) show asymmetric curves with an ambipolar behavior similar to BGTC devices (see output curves in Supplementary Figure 11). We should mention that the *I*_DS_ for the MAPbI_3_-based device is about 10 times higher than that of the Cl- and Br-based devices, which is due to the fact that the channel width (*W*) of each device depended upon the lateral size of the TSCs and was measured individually for each TSC. The figures of merit and the channel size of BGBC devices are summarized in Table 2 (*V*_GS_ scanning direction: from  −10 to 10 V; *V*_DS_ =  −2 V). The maximum linear hole (electron) mobilities of 3.8 (0.32), 3.6 (0.26), and 4.7 (1.51)cm^2^ V^−1^s^−1^ at room temperature were achieved for X = Cl, Br, and I, respectively (Table 2 and Supplementary Figure 12), while the average linear hole (electron) mobilities for the same were 2.1 (0.27), 2.3 (0.19), and 2.9 (1.12)cm^2^V^−1^s^−1^ (Supplementary Table 2). The mobility vs. *V*_GS_ for the FETs based on MAPbCl_3_, MAPbBr_3_, and MAPbI_3_ TSCs is shown in Supplementary Figure 13. At a value of *V*_DS_ =  −2 V, the linear charge mobilities are corresponding to the these shown in Table 2 and Supplementary Table 2. The current on/off ratios are found to be on the order of 10^5^ and *V*_TH_ was found to be typically low (±1.6 V). The variations in the mobility are difficult to explain with the current level of understanding of hybrid perovskites and will require significantly more experimental and theoretical insight. The same can be said for differences in the polarity of devices. Both are realistically linked to the choice of contact material, to processing conditions, and to the quality of the channel. The SC–electrode contact resistance plays a role. Mobility is known to be linked to the quality of the SC–dielectric interface in the channel of FET devices. Hence, the presence of and variations in structural and morphological defects, chemical contaminations, and unreacted reagents are expected to influence carrier mobility.

**Table 2:** The figures of merit of BGBC TSC-FET devices based on MAPbX_3_ (X = Cl, Br, and I)

TSCs	*W*/*L* (µm µm^−1^)	Highest *µ*_h_ (cm^2^ V^−1^s^−1^)	Average *µ*_h_ (cm^2^V^−1^s^−1^)	On/off ratio	*V*_TH_ (V)	*N*_it_ (eV^−1^ cm^−2^)	*S* (V dec^−1^)
MAPbCl_**3**_	20/10	3.8	2.1	1.45 × 10^5^	1.45	3.27 × 10^12^	2.1
MAPbBr_**3**_	20/10	3.6	2.3	3.37 × 10^5^	1.15	3.03 × 10^12^	1.9
MAPbI_**3**_	55/10	4.7	2.9	8.78 × 10^4^	0.16	3.79 × 10^11^	0.6

The state-of-the-art mobility results highlight the overall success and versatility of the confinement crystallization approach and its ability to successfully integrate hybrid perovskite SCs into FET devices even when pre-patterned electrodes are used. The relatively high field-effect mobility measured in hybrid perovskite SC-FET devices at room temperature can be attributed to excellent planarity, absence of grain and domain boundaries, low surface roughness, and reduced chemical contamination at the buried semiconductor–dielectric interface inside the channel. Mitigation of these imperfections through confined crystallization has helped the FET devices tap more easily into the properties of the bulk semiconductor. We have calculated the interfacial trap state density (*N*_it_) from the subthreshold swing, *S*, in the subthreshold regime (Supplementary Figures 14 and 15) using the formulae *S* = d*V*_GS_/d(log *I*_DS_) and *N*_it_ = (*Sq*/*k*_B_*T* ln 10–1)(*C*_i_/*q*), where *q* is the elementary charge, *k*_B_ is the Boltzmann constant, and *T* is the temperature, as summarized in Tables 1 and 2. The calculated *N*_it_ values for MAPbX_3_ TSCs with X = Cl, Br, and I in BGBC (BGTC) device configurations are 3.3 × 10^12^ (5.3 × 10^12^), 3.0 × 10^12^ (4.6 × 10^12^), and 3.8 × 10^11^ (3.7 × 10^12^)eV^−1^cm^−2^. The MAPbI_3_ SC-FET exhibits the lowest *S* of 0.6 and 3.8 Vdec^−1^, respectively, in BGBC and BGTC devices, corresponding to the lowest *N*_it_. The low *N*_it_ values point to the formation of a reasonably high-quality semiconductor–dielectric interface. It should be noted here that the *N*_it_ for the I-based devices were ca. 10 times lower than those of the Cl- and Br-based devices, because of differences in lateral size of the SCs defining the channel width (*W*).

By comparing with the state-of-art FET researches based on perovskite materials (Supplementary Table 3), the field-effect mobility values reported at room temperature for MAPbCl_3_, MAPbBr_3_, and MAPbBr_3_ are the highest to date for FETs made, thus far, with polycrystalline films of these materials^7,11^ and are the first report of single crystal hybrid perovskite FET devices. The hole mobility of MAPbI_3_ perovskites is improved by approximately two- to fivevfold compared with the PTF mobility at room temperature^7^, and is a rough order of magnitude higher than that measured at 77 K^11^. It is also worth noting that the hole mobilities reported herein are competitive with many other solution-processed semiconductors used in *p*-channel FETs, including organics^48^, metal oxides^49^, pseudo halides^50,51^, and quantum dot solids^52^.

Hybrid perovskite semiconductors have recently been identified as mixed ionic–electronic conductors owing to the ability of charged defect ions to migrate under an external bias^53–55^. The resulting effect is a hysteresis in current–voltage sweeps. This effect has been broadly reported for solar cells, and more recently in memristors and light-emitting FETs fabricated using hybrid perovskites^56–58^. The hysteresis effects was speculated to be related to screening effects arising from field-induced drift of methylammonium cations^59^. Charge traps and surface dipoles at the untreated dielectric–semiconductor interface may also be attributed to the hysteresis effect^60^. For BGBC devices, the hysteretic behavior and influence of scan rate and direction on FET devices based on MAPbCl_3_, MAPbBr_3_, and MAPbI_3_ TSCs was studied by sweeping the voltage in transfer curves in the sequence −10 → 0 → + 10 → 0 → −10 V for BGBC devices at rates ranging from 0.05 to 0.25 V s^−1^. The *V*_GS_ forward and reverse scanning directions at a rate of 0.05 V s^−1^ are shown in Supplementary Figure 16. We can find that the carrier mobility extracted from the forward scanning gate sweep is higher than that extracted from the reverse scanning gate sweep, especially in the case of MAPbI_3_. The effect was lower in MAPbCl_3_ and negligible in MAPbBr_3_. The measured current values are plotted on a logarithmic scale in Supplementary Figure 17. The transfer curves and the output curves exhibit weak but notable hysteresis effects (Fig. 4), which is fortunately not substantial in low sweep rates. Moreover, larger open circuit voltages^10^ (the difference of voltage between two current minimum points) and less pronounced hysteresis were detected in conditions of slower sweep rate of the transfer curves. The hysteresis becomes more severe and the saturation current decreased with increasing sweep rate. For instance, significant hysteretic behavior was observed when BGTC devices (Supplementary Figure 18) were deliberately subjected to large *V*_GS_ sweeps (±40 V) and fast sweep rate (0.5 V s^−1^). Defect ion migration is more favorable at slower than at faster sweep rates, since they are less mobile than electronic carriers (electrons and holes), making hysteresis more severe and reducing the saturation current in faster sweep conditions.

## Discussion

We have demonstrated high-performance FET devices based on MAPbX_3_ (X = Cl, Br, and I) hybrid perovskite single crystals both in BGTC and BGBC device configurations. The confined crystal growth approach was found to be crucial for the successful integration of hybrid perovskite SCs into FET devices, a class of devices that has been particularly challenging to fabricate and operate for MAPbX_3_ semiconductors. In particular, the spatial confinement during the inverse temperature crystallization process produced topographically ultra-smooth top and bottom facets free of grains or domain boundaries and any other corrugations typically observed in free-grown crystals and polycrystalline films. This is credited with producing high-quality semiconductor–dielectric interfaces in the channel, free of common morphological defects and chemical impurities often found on the surface of hybrid perovskite SC polycrystalline films. This approach also eliminates grain boundaries common in polycrystalline films and known to be a source of resistance to lateral transport due to tunnel junction formation. Through vertical confinement, the crystals also easily grew laterally, allowing them to reach millimeter scale or larger and readily bridged FET channels ranging in length from 10 to 150μm. Use of a pre-patterned substrate with metal electrodes furthermore promoted conformal growth of the TSCs into the channel and formed high-quality semiconductor–dielectric interface as well as electrical contacts with strong adhesion. The on/off current ratio in the 10^3^–10^5^ range was readily achieved at room temperature. Furthermore, the devices showed low operating voltages (<5 V) with the lowest (<2 V) voltages observed in BCBG devices. Overall, the maximum room temperature hole (electron) mobility ranged from 2.6 to 4.7 (0.26–2.2)cm^2^V^−1^s^−1^ across the different halides and device configurations, with average hole (electron) mobility in the 1.5–2.9 (0.19–1.3)cm^2^V^−1^s^−1^ range, significantly outperforming thin film-based MAPbI_3_ FET devices reported to date (Supplementary Table 3). During the revision of this paper, a relevant article to the topic of perovskite FETs was published.^61^ Demonstration of transparent MAPbCl_3_ SCs yielding field-effect saturation hole (electron) mobility as high as 2.6 (2.2)cm^2^V^−1^s^−1^ makes this a potentially interesting material candidate for transparent and imperceptible electronics. Overall, this work demonstrates the feasibility of solution-grown organohalide perovskite FET devices by addressing interfaces and contacts. This makes hybrid perovskites a viable material platform for printed and transparent electronics.

## Methods

### Chemicals

The CH_3_NH_3_X (X = Cl, Br, and I) were purchased from Dyesol and used as received. Lead halide PbX_2_ (X = Cl, Br, and I) dimethylformamide (DMF) and γ-butyrolactone (GBL) were purchased from Sigma-Aldrich and used as received. PET film (2.5 µm thick) was purchased from 2spi.com.

### TSC synthesis

The substrate (glass or Si/SiO_2_ pre-patterned with Au electrodes for FET fabrication) and superstrate (glass) with dimensions of 1.5 cm × 1.5 cm were cleaned by rinsing with acetone, iso-proponal, and deionized water in turn. After drying in vacuum oven overnight, the substrates were exposed to UV-O_3_ for 10 min. Subsequently, two PET strips were attached to the substrate with a lateral spacing of several millimeters. The superstrate was placed on top and the sandwiched stack was heated to 270 °C on a hot plate for 5 min to melt the PET and help adhere the substrate and the superstrate. The substrates were transferred upon cooling to a nitrogen glove box (below 0.1ppm O_2_ and 0.1ppm H_2_O). The hybrid perovskite MAPbX_3_ (X = Cl, Br, and I) were resolved in DMF, DMF, and GBL, respectively, at 1 M concentration to prepare the precursor solution in the glove box. A small volume (6 µl) of precursor solution was then injected at the edge of the sandwich and was drawn and spread into the gap through capillary force. Crystal growth was achieved by placing the stack on a hot plate and annealing at a fixed temperature of 60 °C (2 days), 80 °C (2 days), and 100 °C (3 days) for MAPbCl_3_, MAPbBr_3_, and MAPbI_3_, respectively.

### Characterization

UV–Vis absorbance spectra were measured using a Cary 5000 (Varian) spectrophotometer equipped with an integrating sphere. Photoluminescence measurements were conducted on a DXR Smart Raman spectrometer using the excitation lasers at 325 and 473 nm. Fluorescence microscopy measurements were performed in Zeiss LSM 710 Upright Confocal Microscope. Optical micrographs were acquired using a Nikon SMZ25 stereomicroscope. Film and crystal thickness were measured by a DEKTAK 8 profilometer (Veeco). XRD measurements were performed at room temperature using a D8 Discover X-ray diffractometer (Bruker). XRC was measured by Bruker D8 Advance using a wavelength of 1.54 Å and LynxEye XE-T detector with 0D mode. SEM was performed using an FEI Nova Nano 630. 2D SEM was taken on a Phenom ProX system. STM characterization was executed in UHV conditions (5.0 × 10^−10^ mbar) in a variable temperature STM (VT-STM; Omicron Nanotechnology). Chemically etched polycrystalline tungsten wire was used as STM tip for imaging, which was further cleaned by electron bombardment in situ in UHV to reach atomically resolved imaging of HOPG. As-grown SC samples on conducting substrate (ITO) were mounted on a sample plate for STM studies. All images were acquired with a tunneling parameter of 2.4 V, 0.6 nA. For XPS measurements, the as-grown samples were grown and sealed in a vial in N_2_-filled glove box, and quickly transferred to the XPS vacuum chamber. XPS studies were carried out using a Kratos Axis Ultra DLD spectrometer equipped with amonochromatic Al Ka X-ray source (*hν* = 1486.6 eV) operating at 150 W, as well as a multichannel plate and a delay line detector under a vacuum pressure of 1 × 10^−9^ mbar. Measurements were performed in hybrid mode using electrostatic and magnetic lenses, and the take-off angle (the angle between the sample surface normal and the electron optical axis of the spectrometer) was 0°. Binding energies were referenced to the C 1s peak of the (C–C, C–H) bond, which was set to 285.0 eV. The data were analyzed using the commercially available software program CASAXPS.

### FET fabrication and characterization

For BGBC FET devices, the Si/SiO_2_ substrates (capacitance *C*_i_ of 15nFcm^−2^) with gold pattern were used as substrates. For BGTC FET devices, perovskite TSCs were first grown on the Si/SiO_2_ substrates, and then 80 nm Au electrode was thermally evaporated on top with masks. Different lengths (10, 20, 50, 100, and 150 μm) were utilized. The channel width (*W*) depends upon the lateral size of the TSC and was measured individually for each TSC that was successfully grown on a pair of FET device electrodes. *I–V* measurements were performed at room temperature using a Keithley 4200 Semiconductor Parametric Analyzer and a Signotone Micromanipulator S-1160 probe station in continuous mode. The measuring process was conducted under vacuum.

## Supplementary information

Supplementary Information

## Data Availability

All data needed to evaluate the conclusions in the paper are present in the paper and/or the Supplementary Materials. Additional data related to this paper may be requested from the authors.
