# Supplementary material for: Single crystal hybrid perovskite field-effect transistors
Source: Nat Commun. 2018 Dec 17;9:5354. doi: 10.1038/s41467-018-07706-9 (PMC6297354; doi:10.1038/s41467-018-07706-9)
Supplement: Supplementary file 1 — Supplementary Information [file 41467_2018_7706_MOESM1_ESM.pdf]

Single-crystal hybrid perovskite field-effect transistors

Yu et al.

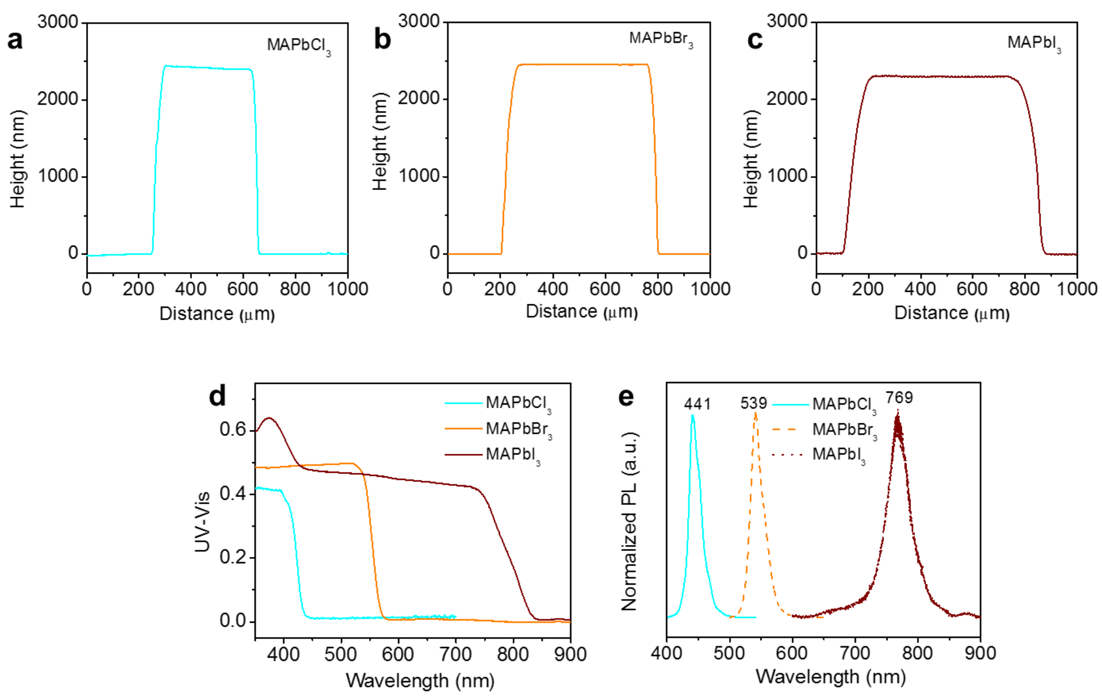

Supplementary Figure 1. Thickness, absorbance and PL measurements. (**a**)-(**c**) The thickness profile of typical large $MAPbCl_3$, $MAPbBr_3$ and $MAPbI_3$ thin SCs, respectively. (**d**) UV-Vis absorbance spectra and (**e**) steady state photoluminescence spectra of $MAPbX_3$ (X = Cl, Br, I) thin SCs are shown.

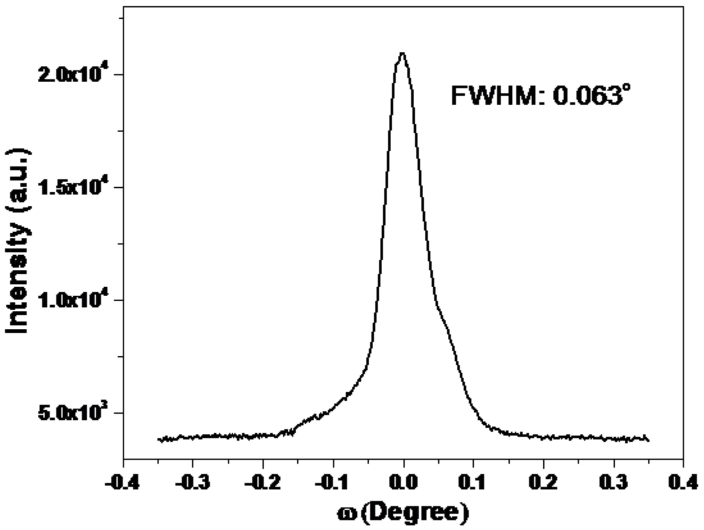

Supplementary Figure 2. The X-ray diffraction rocking curve (XRC) thin $MAPbBr_3$ thin SCs.

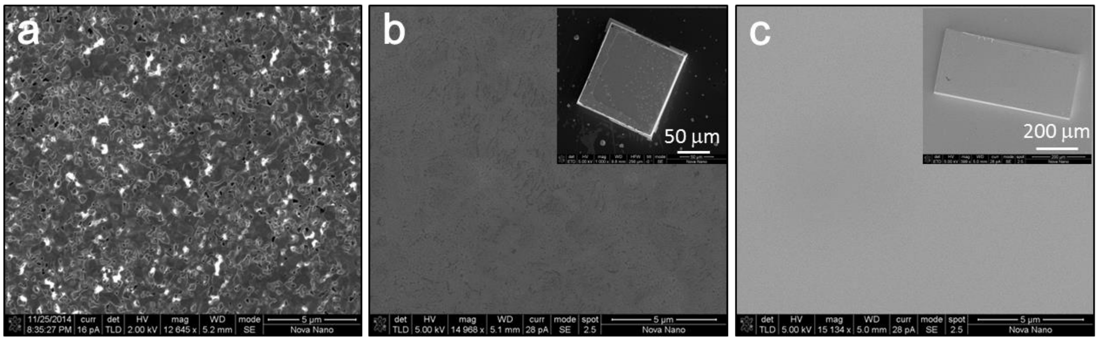

Supplementary Figure 3. SEM comparison of surface morphology. SEM images of (**a**) a polycrystalline thin film, (**b**) a bulk SC and (**c**) a thin SC of $MAPbBr_3$. The SEM images confirm that the surface of the thin SC is much smoother and uniform in appearance than either the bulk single crystal or thin film.

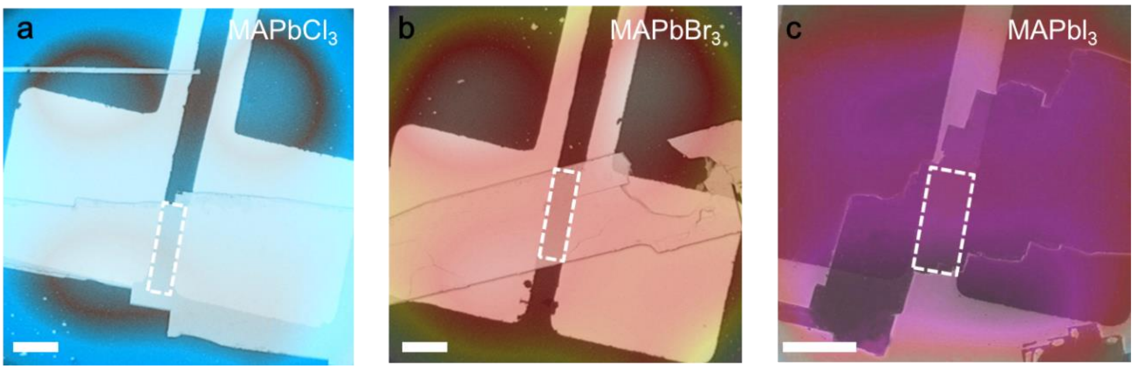

Supplementary Figure 4. 2D false colour SEM micrographs of the perovskite BGTC devices for (a) $MAPbCl_3$, (b) $MAPbBr_3$ and (c) $MAPbI_3$. The width and length for $MAPbCl_3$ are 265 $\times$ 50 µm as shown below. For $MAPbBr_3$, the width and length are 240 $\times$ 50 µm. For $MAPbI_3$, the width and length are 185 $\times$ 50 µm. The scale bar is 100 µm.

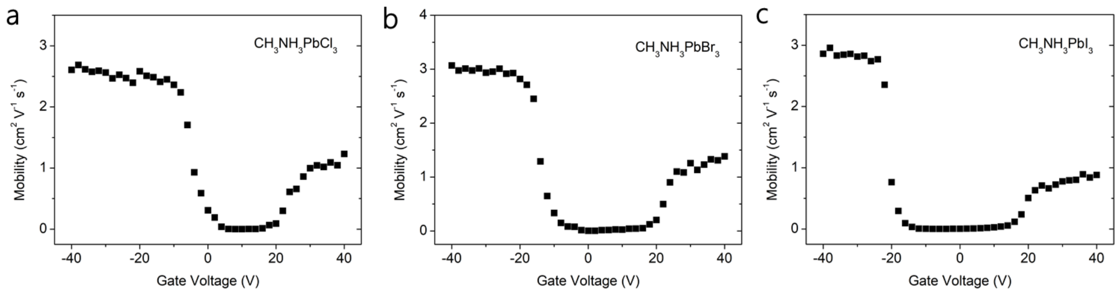

Supplementary Figure 5. The saturation field-effect mobility *vs* gate voltage of (**a**) $MAPbCl_3$, (**b**) $MAPbBr_3$ and (**c**) $MAPbI_3$ FETs employing BGTC configuration.

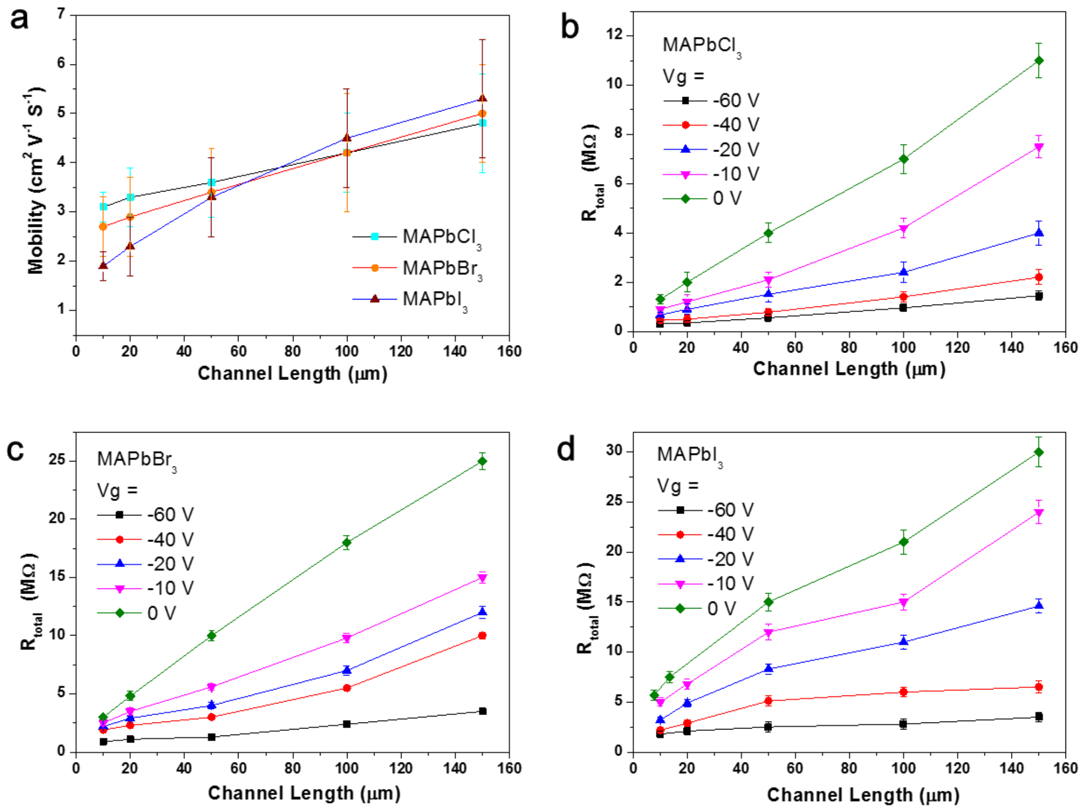

Supplementary Figure 6. (a) Mobility and (b-d) Total resistance versus channel length for different perovskite thin SCs. Structure: BGTC. V_{ds} is -10 V. The contact resistance of various perovskite thin SCs was achieved following the Transmission Line Mode, $R_{total} = R_{channel} + R_c = L\rho_{channel} + R_c$, where R_{total} is the total resistance, L is channel length, and R_c is contact resistance. From (**b**)-(**d**), the R_c for all perovskites can be determined from extrapolation to L = 0. Dozens of samples with different channel lengths were utilized to determine the R_c against for gate voltages. The total device resistance R_{total} multiplied by the channel width W is calculated to be 80 kΩ cm, 150 kΩ cm and 195 kΩ cm for $MAPbCl_3$, $MAPbBr_3$, $MAPbI_3$, respectively. This allows us to compare the different materials directly. (L. Bürgi, T. J. Richards, R. H. Friend and H. Sirringhaus, *Journal of Applied Physics*, 2003, 94, 6129-6137.)

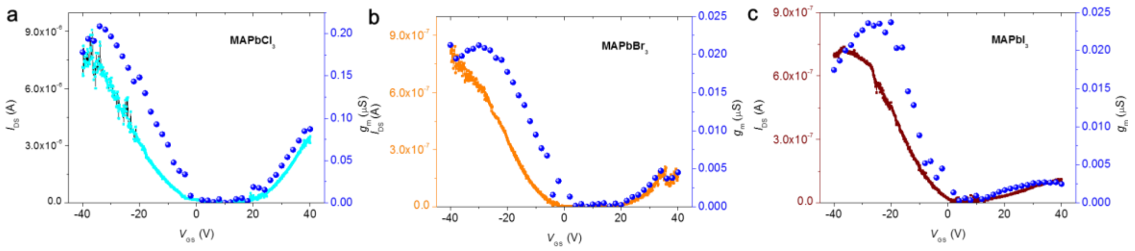

Supplementary Figure 7. The transfer curve (solid) and trans-conductance (dotted) for the BGTC devices of (**a**) $MAPbCl_3$, (**b**) $MAPbBr_3$ and (**c**) $MAPbI_3$.

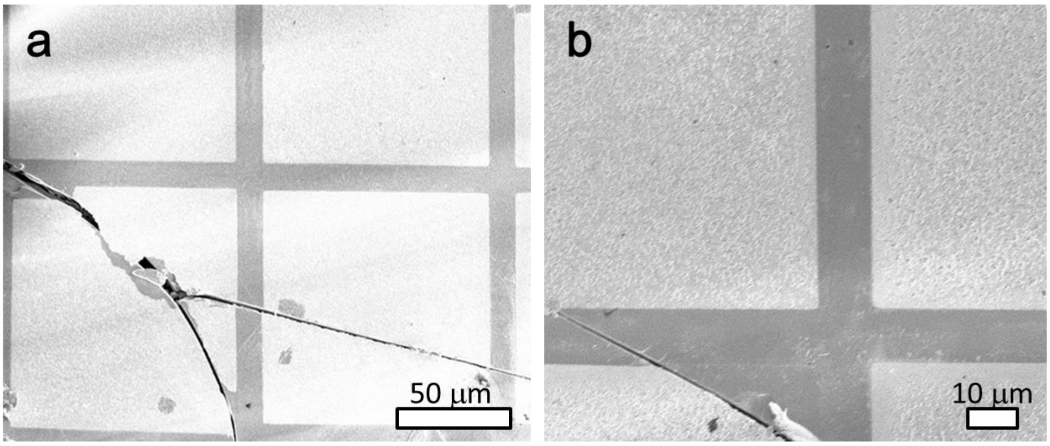

Supplementary Figure 8. SEM characterization of TSC bottom surface morphology of BGBC devices. SEM images of (**a**) bottom surface of TSC with Au electrode, (**b**) enlarged SEM image. The SEM images confirm that the surface of the TSC is much smoother and uniform in appearance than either the bulk single crystal or thin film.

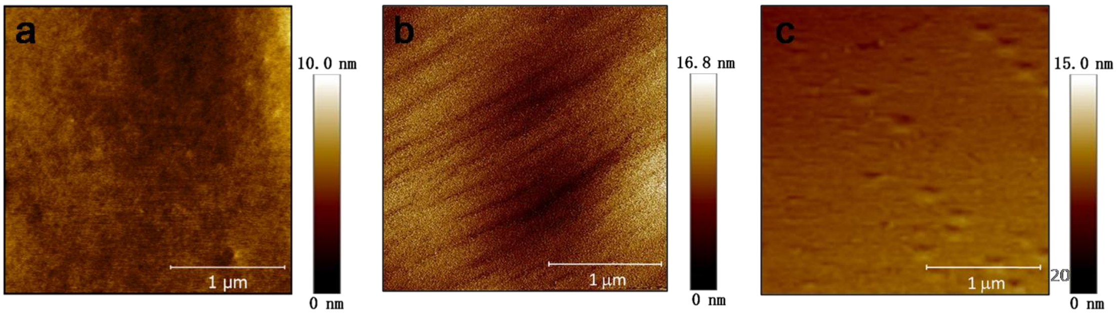

Supplementary Figure 9. AFM characterization of TSC bottom surface. The RMS values of the roughness of a) $MAPbCl_3$, b) $MAPbBr_3$ and c) $MAPbI_3$ are 0.70 nm, 1.23 nm and 1.02 nm, respectively.

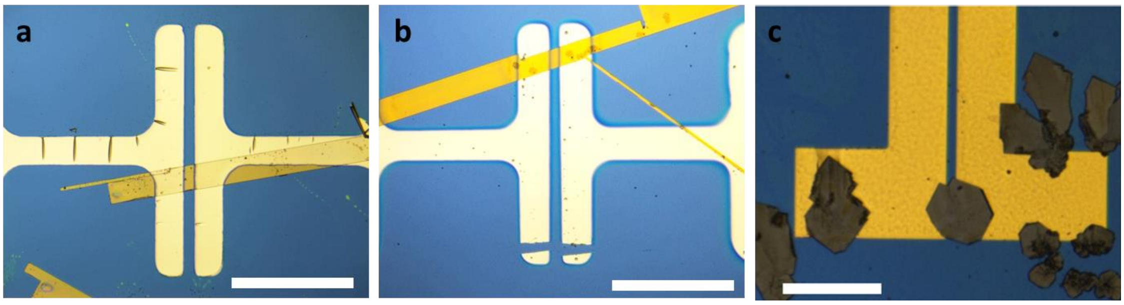

Supplementary Figure 10. Typical optical micrographs of BGBC thin SC-FETs. Optical images of thin SC-FET devices of (**a**) $MAPbCl_3$, (**b**) $MAPbBr_3$ and (**c**) $MAPbI_3$ thin SCs fabricated on Si/SiO_2 substrates pre-patterned with Au electrodes. Scale bar: 100 µm.

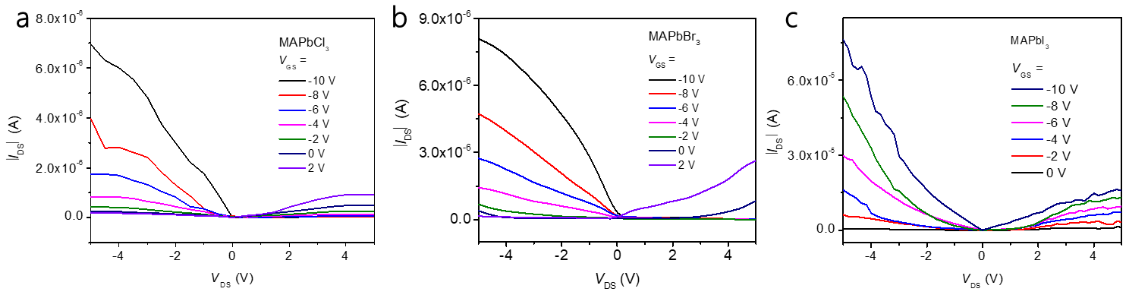

Supplementary Figure 11. Output characteristics of BGBC devices. Output characteristic of (**a**) $MAPbCl_3$, (**b**) $MAPbBr_3$ and (**c**) $MAPbI_3$ thin SCs.

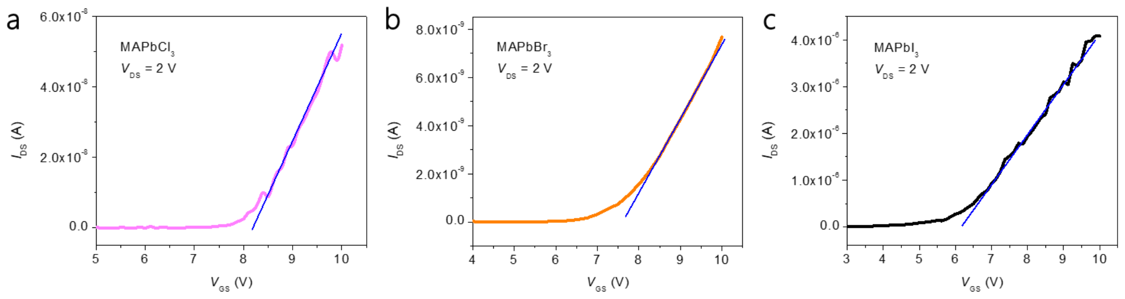

Supplementary Figure 12. Transfer characteristics of BGBC devices. Transfer characteristics of (**a**) $MAPbCl_3$, (**b**) $MAPbBr_3$ and (**c**) $MAPbI_3$ thin SC-FETs, respectively, for electron mobility calculation.

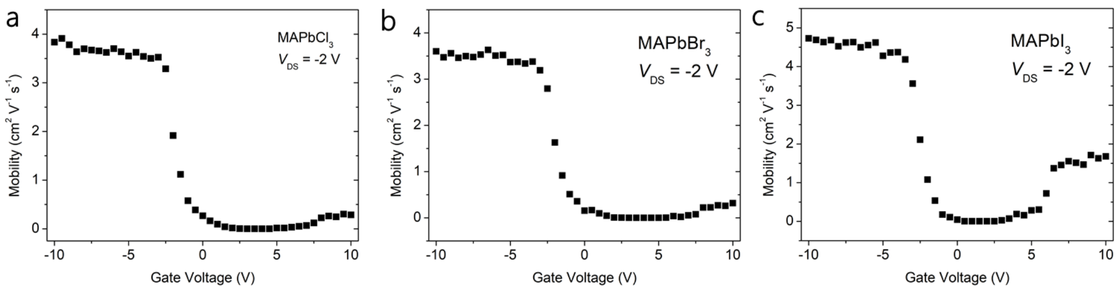

Supplementary Figure 13. The field-effect mobility *vs* gate voltage of (**a**) $MAPbCl_3$, (**b**) $MAPbBr_3$ and (**c**) $MAPbI_3$ FETs employing BGBC configuration.

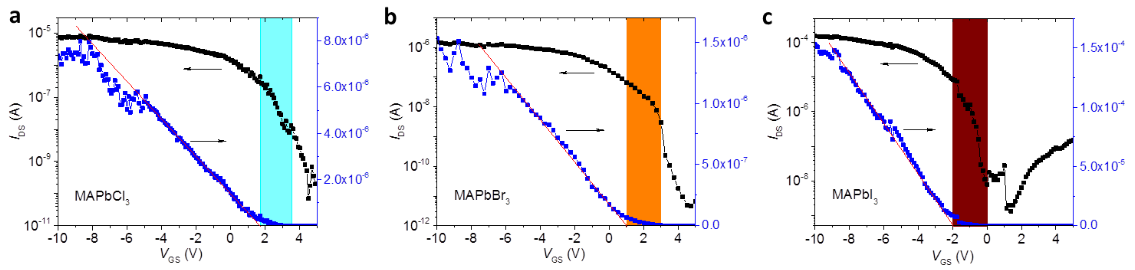

Supplementary Figure 14. Log-scale transfer characteristics for BGBC devices. Log scale transfer characteristic of (**a**) $MAPbCl_3$, (**b**) $MAPbBr_3$ and (**c**) $MAPbI_3$ thin SCs, highlighting the subthreshold regime.

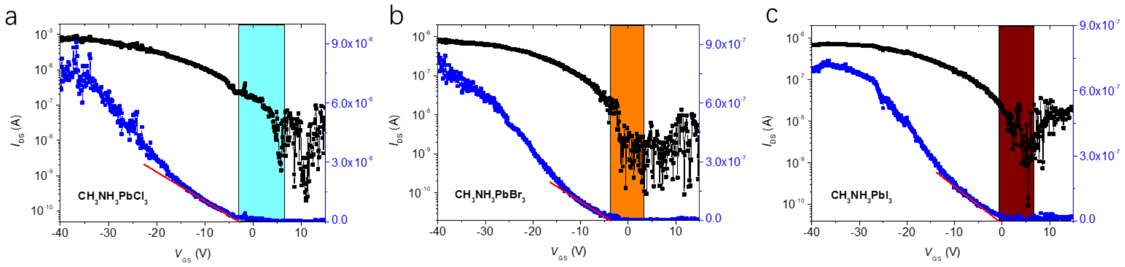

Supplementary Figure 15. Log-scale transfer characteristics for BGTC devices. Log scale transfer characteristic of (**a**) $MAPbCl_3$, (**b**) $MAPbBr_3$ and (**c**) $MAPbI_3$ thin SCs, highlighting the subthreshold regime.

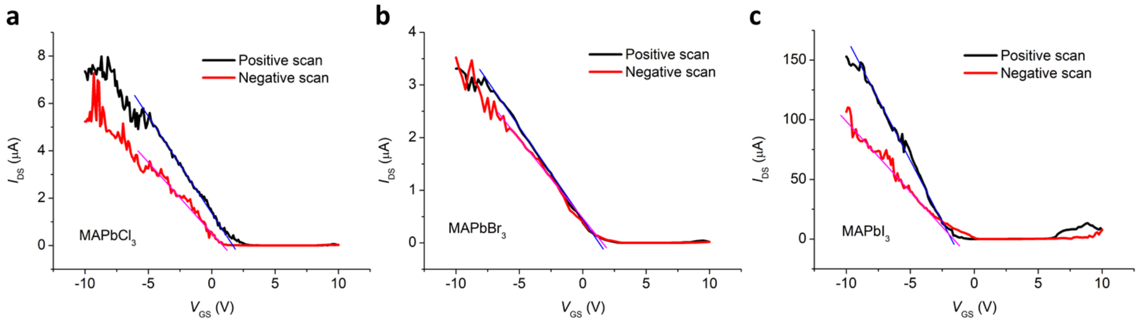

Supplementary Figure 16. Transfer characteristics with different gate voltage scanning directions for BGBC devices. (**a**) $MAPbCl_3$, (**b**) $MAPbBr_3$ and (**c**) $MAPbI_3$ thin SC-FETs, respectively. The gate-source voltage scans between 10 V to -10 V at a rate of 0.05 V/s for hole mobility calculation. This figure exhibited the hysteresis effect of the BGBC devices. The field effect mobility extracted from the forward gate sweep is higher than that extracted from the backward gate sweep. The forward (positive) gate sweep field effect mobility for $MAPbCl_3$, $MAPbBr_3$ and $MAPbI_3$ are 3.8, 3.6 and 4.7 $cm^2 V^{-1} s^{-1}$, respectively, while the reverse (negative) gate sweep field effect mobility for $MAPbCl_3$, $MAPbBr_3$ and $MAPbI_3$ are 3.65, 3.57 and 4.23 $cm^2 V^{-1} s^{-1}$, respectively.

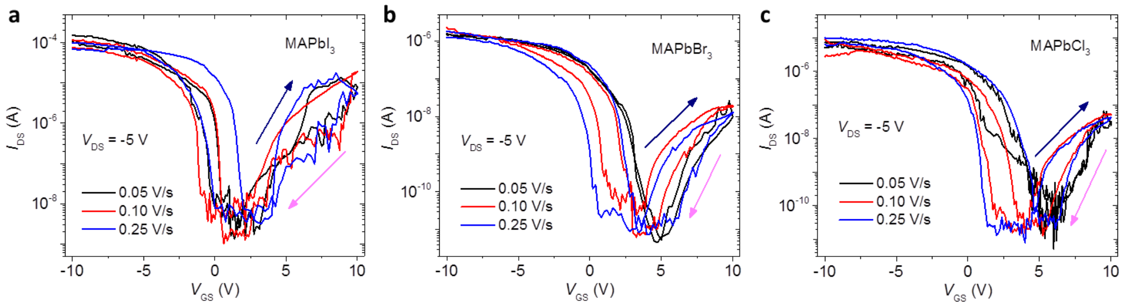

Supplementary Figure 17. Transfer curves with different voltage sweep directions and rates. Transfer characteristics of the field-effect transistors based on (**a**) $MAPbCl_3$, (**b**) $MAPbBr_3$ and (**c**) $MAPbI_3$ thin SCs under different scanning rates from 0.05 Vs^{-1} to 0.25 V s^{-1}, and different directions.

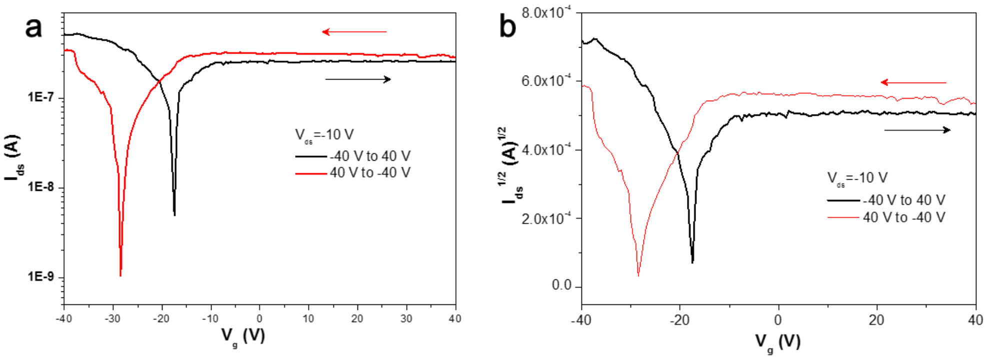

Supplementary Figure 18. Transfer curves for BGTC structured $MAPbI_3$ TSCs devices. L = 100 μm, W = 400 μm. V_g scan from -40 V to 40 V and from 40 V to -40 V. Scan rate: 0.5 Vs^{-1}.

Supplementary Table 1. The figures of merit of BGTC thin SC-FET devices.

Thin SCs	Highest μ_h ($cm^2 V^{-1} s^{-1}$)	Average μ_h ($cm^2 V^{-1} s^{-1}$)	μ_h standard deviations ($cm^2 V^{-1} s^{-1}$)	Highest μ_e ($cm^2 V^{-1} s^{-1}$)	Average μ_e ($cm^2 V^{-1} s^{-1}$)	μ_e standard deviations ($cm^2 V^{-1} s^{-1}$)
**$MAPbCl_3$**	2.6	1.8	0.6	2.2	1.3	0.5
**$MAPbBr_3$**	3.1	1.9	0.7	1.8	1.1	0.4
**$MAPbI_3$**	2.9	1.5	0.7	1.1	0.7	0.3

Supplementary Table 2. The figures of merit of BGBC thin SC-FET devices.

Thin SCs	Highest μ_h ($cm^2 V^{-1} s^{-1}$)	Average μ_h ($cm^2 V^{-1} s^{-1}$)	μ_h standard deviations ($cm^2 V^{-1} s^{-1}$)	Highest μ_e ($cm^2 V^{-1} s^{-1}$)	Average μ_e ($cm^2 V^{-1} s^{-1}$)	μ_e standard deviations ($cm^2 V^{-1} s^{-1}$)
**$MAPbCl_3$**	3.8	2.1	0.9	0.32	0.27	0.04
**$MAPbBr_3$**	3.6	2.3	0.7	0.26	0.19	0.05
**$MAPbI_3$**	4.7	2.9	1.0	1.51	1.12	0.12

Supplementary Table 3. The state-of-art FET mobilities reported from hybrid perovskite semiconductors.

Material	μ_h (cm^2 V^{-1} s^{-1}) & Temperature	μ_e (cm^2 V^{-1} s^{-1}) & Temperature	Reference
$CH_3NH_3PbI_3$	6.6×10^{-3} ($\mu_{h,linear}$); 2.1×10^{-2} ($\mu_{h,saturation}$) at 78 K	6.7×10^{-2} ($\mu_{e,linear}$); 7.2×10^{-2} ($\mu_{e,saturation}$) at 78 K	Nat Commun 2015, 6, 7383
$CH_3NH_3PbI_3$	0.18 at RT[a]	0.17 at RT	Nat Commun 2015, 6, 8238
$CH_3NH_3PbI_{3-x}Cl_x$	1.3 at RT	1.0 at RT	MRS Commun 2015, 5, 297
$CH_3NH_3PbI_3$	--	>1 at 77 K	Science Adv 2015, 1, e1500613
$CH_3NH_3PbI_3$	--	0.5 at 160 K	Nat Commun 2016, 7, 11330
$(C_6H_5C_2H_4NH_3)_2SnI_4$	15 at RT	--	Adv Mater 2016, 28, 10275
$Cs_x(MA_{0.17}FA_{0.83})_{1-x}Pb(Br_{0.17}I_{0.83})_3$	2.02 at RT	2.39 at RT	Adv Mater 2017, 29，1602940
$CH_3NH_3PbI_3$	>1 at a few K	3 at 160 K	Adv Mater 2017, 29, 1601959
$CH_3NH_3PbI_3$	--	0.05 cm^2/Vs at RT[b] >2 cm^2/Vs at 100 K	Science Adv 2017, 3, e1601935
$MAPbI_3$	>10 at RT	>10 at RT	Adv Electron Mater 2018, 1800316

Note: [a] RT means room temperature. [b] champion mobility of 3 cm^2/Vs after modifying the contact with polyethylenimine ethoxylate (PEIE; ~2 nm thick).
